# Detecting Sensitive Spectral Bands and Vegetation Indices for Potato Yield Using Handheld Spectroradiometer Data

**DOI:** 10.3390/plants13233436

**Published:** 2024-12-07

**Authors:** Diego Gomez, Pablo Salvador, Juan Fernando Rodrigo, Jorge Gil

**Affiliations:** 1 Joint Research Centre (JRC), European Commission, 21027 Ispra, Italy; 2 Council of Science and Education, Castilla and Leon Regional Government, 47014 Valladolid, Spain; 3 Independent Researcher, 47007 Valladolid, Spain

**Keywords:** food security, potato crop, precision agriculture, spectrometer instrument

## Abstract

Remote sensing is a valuable tool in precision agriculture due to its spatial and temporal coverage, non-destructive method of data collection, and cost-effectiveness. In this study, we measured the canopy reflectance of potato (*Solanum tuberosum* L.) crops on a plant-by-plant basis with a handheld spectrometer instrument. Our study pursues two primary objectives: (1) determining the optimal temporal aggregation for measuring canopy signals related to potato yield and (2) identifying the best spectral bands in the 350–2500 nm domain and vegetation indices. The study was conducted over two consecutive years (2020 and 2021) with 60 plants per plot, encompassing six potato varieties and three replicates annually throughout the growth season. Employing correlation analysis and dimensionality reduction, we identified 23 independent features significantly correlated with tuber yield. We used multiple linear regression analysis to model the relationship between the selected features and yield and to compare their influence in the fitted model. We used the Leave-One-Out Cross-Validation (LOOCV) method to assess the validity of the model (RMSE = 702 g and %RMSE = 29.2%). The most significant features included the Gitelson2 and Vogelmann indices. The optimal time period for measurements was determined to be from 56 to 100 days after planting. These findings may contribute to the advancement of precision farming by proposing tailored sensor applications, paving the way for improved agricultural practices and enhanced food security.

## 1. Introduction

Current population growth, the impact of climate change, and geopolitical conflicts notably alter food prices and availability, exerting high pressure on agriculture and natural resources [1]. Global food demand is estimated to rise from 35% to 56% between 2010 and 2050, with a subsequent change in the population at risk of hunger [2]. In this context, food production faces a critical challenge that binds together a net increase in food supply, fewer resources, fewer negative environmental impacts, and the need to avoid augmenting inequalities [1].

Potato (*Solanum tuberosum* L.) is one of the most important non-grain foods worldwide, with a total production of 359 million tons [3]. It plays an important role in food security, being a source of vitamin C and B6, fiber, sodium carbs, and other micronutrients, and it is fat-free [4]. Potato crops are spread over 150 countries from latitudes 69° N to 50° S [5], which implies that they are either very adaptable crops or are grown under sub-optimal conditions in many places [6]. Despite this dichotomy, spatial and temporal crop yield variability is of great concern among farmers, the food industry, and decision/policymakers, with an existing gap between yield potential and actual yields [7,8]. Soil constraints, weather variability, agricultural management, crop pests, and genetic factors (i.e., varieties) account for the largest sources of crop yield variability [9,10,11]. In general, crop-growth models include these factors to assist with agricultural practices and yield estimations. Current efforts focus on the integration of remote sensing data with crop growth models to blend the advantages of both methods [12,13]. Earth observation data allows us to monitor crop status over time and space in a cost-effective manner by active (Synthetic Aperture Radar) and passive (Optical) remote sensing systems. The data is acquired at a distance (from a few centimeters—field portable spectroradiometers—to hundreds of km—satellites) by a sensor that measures the received radiation. Historical records, economic costs, and spatial/temporal resolutions are the main characteristics used to decide which remote sensing platform to use (with satellite data being the most widely used). Nevertheless, the multispectral sensors that are onboard most satellites have specific spectral bands and widths that may not work well for very specific cases. Thus, hyperspectral spectro-radiometers are convenient tools for determining which bands in the electromagnetic spectrum are more sensitive to derive specific characteristics of the target object (e.g., chlorophyll, nitrogen). However, this entails some challenges, such as multicollinearity among adjacent spectral bands, resulting in information redundancy [14]. Exhaustive in-situ measurements may allow tailoring sensors for particular objectives/crops.

Previous works have investigated the potential of hyperspectral data to evaluate crop-growth status (e.g., aboveground biomass, chlorophyll, nitrogen content) and yield across different crops, including winter wheat [15], rice [16], maize [17], and soybean [18]. In potato crops, previous studies used hyperspectral data to detect late light and Alternaria diseases [19,20,21], measure sugar content in different cultivars [22], assess water stress [23], and determine nitrogen content based on different application rates [24,25]. To the best of our knowledge, there has not yet been any study that relates hyperspectral data and crop yield across different varieties and years.

The objectives of this study were to (1) establish the most effective time intervals for recording canopy reflectance data that correlate with potato yield and (2) pinpoint the most predictive spectral wavelengths within the 350–2500 nm range and the derived vegetation indices (VIs) for estimating yield. The term ‘vegetation indices’ refers to mathematical combinations of spectral band reflectances designed to highlight specific properties of plant canopies. The experiment spanned two growing seasons, 2020 and 2021, and was structured into plots, each containing 60 individual potato plants. Hyperspectral data were captured using a portable ASD FieldSpec 3 spectrometer during key growth stages of the potato plants.

## 2. Materials

### 2.1. Study Design

**Figure 1:**
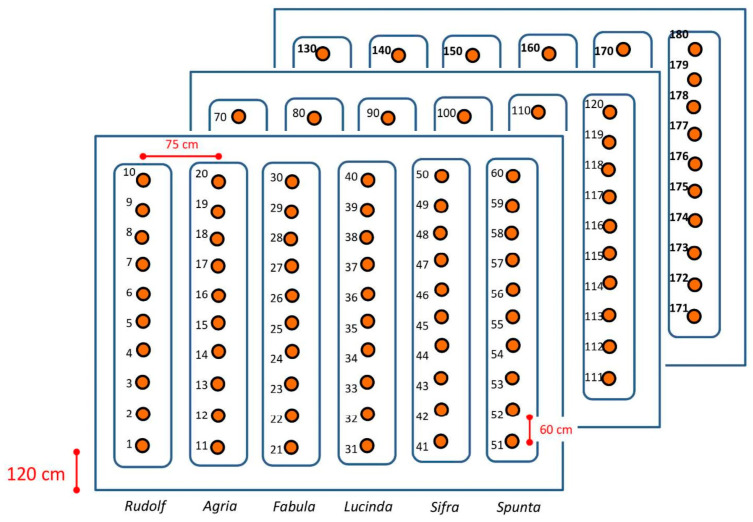
Annual plot layout for the experimental study, showing three separate plots per year. Each plot consists of six rows, one for each potato variety, with 10 plants per row. This results in a total of 60 plants per plot and 180 plants across all three plots for the year.

**Figure 2:**
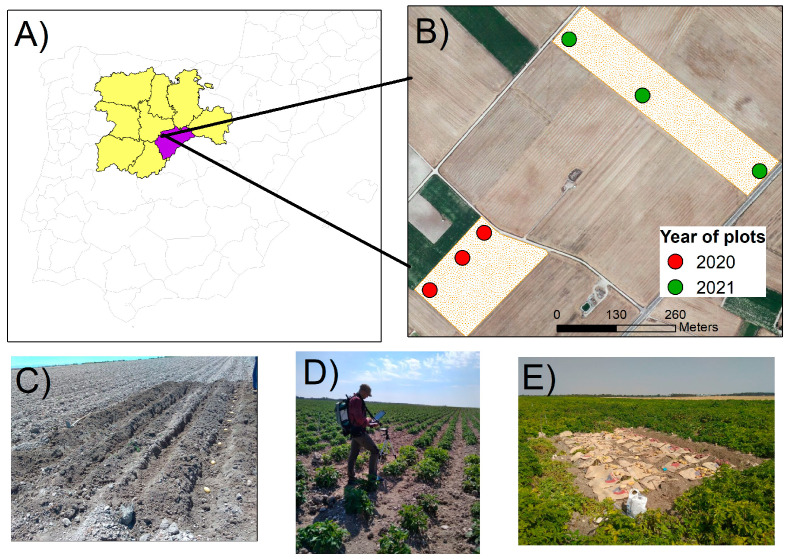
Location of the fields and plots in the study area. The upper image shows the location of Castilla y Leon (yellow) within Spain, with the province of Segovia (purple). (**A**) corresponds to the plot location (red) for the year 2020 and (**B**) for the year 2021. The (**C**–**E**) corresponds to photos taken during the sowing, ASD measurement, and harvest, respectively.

A two-year study was conducted within two commercial farms in the Castilla y León region (Spain). The study area has a Mediterranean cool-dry summer climate (Csb), characterized by dry and hot summers and cold and rainy winters [26]. Fields were located on clay loam soils, and crops were cultivated under a ridge and furrow distribution. The farmer managed fertilization, pest control, and irrigation (by sprinklers) based on crop needs and field scale, including the experimental plots. Within each selected field, we established three experimental plots, each measuring 6 m wide by 8 m long. With three plots per year, each containing 60 plants (six potato varieties with 10 plants per variety), a total of 180 plants were sampled per year, resulting in an overall total of 360 plants across the two-year study. The experimental design, including the number and distribution of plants, was maintained consistently across both years of the study. The layout of the plots and their respective locations in the fields for each year are depicted in Figure 1 and Figure 2. We used six medium-late maturity potato cultivars in the plots: Spunta, Sifra, Lucinda, Fabula, Agria, and Rudolf. Table 1 summarizes dates and characteristics related to planting, harvesting and in situ ASD measurements. Spectral data retrieval was planned at equal time intervals (10–15 days) throughout the growing cycle of the crop. Spectral measurement started when plants had a ground coverage of about 25 cm in diameter (refer to Section 2.2 for further details) until senescence. Due to farming management practices (e.g., irrigation) and atmospheric conditions, these dates had to be adjusted to the local circumstances; hence, the frequency of measurements slightly varied within and between the years (Table 1). The main phenological stages (e.g., closure, flowering, bulking, senescence) were covered by the measurements.

### 2.2. Reflectance Measurements

Spectral reflectance was obtained using a portable ASD FieldSpec 3 spectroradiometer (ASD Inc., Boulder, USA). It operates in the 350–2500 nm spectral region, with a sampling interval of 1.4 nm (350–1050 nm) and 2 nm (1000–2500 nm). The spectral resolution is 3 nm at 700 nm and 10 nm at 1400/2100 nm. A standard whiteboard Spectralon (Labsphere, North Sutton, NH, USA) was used to perform real-time reflectance measurements and optimize the spectral response (re-measured every 5 min).

Plant canopy reflectances were measured on a plant-by-plant basis under clear-sky conditions, within two hours of local solar noon, using a 25° Field-Of-View (bare fiber). The pistol was held perpendicular to the center of each plant (top leaves of canopy) at a constant distance of 30 cm (25° FOV; diameter 14 cm). Five spectral measurements were acquired per plant and then averaged. Thus, we considered one representative spectrum per plant. Repeated measurements were taken throughout the plant life cycle to have spectral information for each growth stage after emergence (Table 1). To diminish soil influence, the first measurement of the year was taken when plants covered at least 25 cm in diameter. This setup allowed us to make a one-on-one comparison across individual plants.

The ASD binary files were converted to ASCII reflectance files using the View Spec Pro software v6.20 (ASD Inc., Boulder, CO, USA) and post-processed to filter out reflectance data in the water absorption bands (1350–1460 and 1790–2000 nm) and to remove noise at 2350–2500 nm [27]. We used R software (v4.1.3) [28] for further analysis.

### 2.3. Tuber Data

Due to the different maturity types across varieties, the harvest date was decided based on the following criteria: (1) >30% of plant vines being dead and brown in a plot, and (2) a similar pattern being observed across plots for the year. Harvesting was meticulously performed on a plant-by-plant basis over three consecutive days (112–114 DAP in 2020 and 125–127 DAP in the year 2021) to deal with tuber collection, transportation, washing, and measurements for the three plots available each year. Each potato plant was harvested individually, and all tubers, regardless of size or malformations, were collected. The weight measurement reported in this study refers to the total weight of tubers harvested from each individual plant. These tubers were then bagged, washed, dried, and weighed, ensuring accurate tracking from harvest to final weight assessment. The individual plant weight, along with the number of tubers and their size categorization, was recorded for analysis with their corresponding IDs (as presented in Figure 1).

Tubers were soaked in cold, fresh tap water and manually washed off to remove adhering soil. Tubers were dried in a room at 20–25 °C without any assisted ventilation method for 12–15 h. Three different measurements were taken on a plant basis: (1) overall tuber weight, (2) number of tubers, and (3) number of tubers categorized into the following calibers: >75 mm, 75–40 mm, or <40 mm. The number and caliber were measured only for contextualization to explore variability across varieties and years. The potato caliber is the transverse diameter of the tuber and was measured using potato sizing squares of dimensions 75 × 75 mm and 40 × 40 mm to assess size and shape. The weight was measured using a digital crane scale with a maximum weight limit of 50 kg and an uncertainty of 0.05 kg. Additionally, we recorded if a plant was impacted by rodents (e.g., marks in tubers, vine damage, etc.). Plants with any sign of tuber damage due to rodents were excluded from the analysis (97 samples).

## 3. Methods

We used the information provided by the individual spectral bands from the ASD instrument to calculate 115 vegetation indices (VI) using the libraries hyperSpec [29] and hsdar [30]. Further information regarding VIs and references can be found at https://rdrr.io/cran/hsdar/man/vegindex.html (accessed on 15 January 2024). Additionally, we included the Potato Productivity Index (PPI) due to the relevance of the crop under analysis [31].

We explored the relationship between canopy reflectance and tuber yield at the plant level using 1678 spectral bands and 116 VIs from each measurement day (d). Aiming for consistency, measurement days were matched as closely as possible in terms of days after planting (DAP) for both study years. We aggregated spectral data using a forward selection method across 21 time-span combinations, which could comprise either single measurement days (e.g., d1, d3) or aggregated measurements across all possible consecutive measurement days (e.g., d1 to d5, d3 to d6), thereby allowing us to evaluate the predictive power of both individual days and cumulative periods, excluding d7 since it was absent for the year 2020. This approach allowed us to evaluate the collective influence of consecutive measurement days on yield prediction, using actual spectral values for single days and combined statistical measures (e.g., mean) for multiple days. This methodical approach aimed to pinpoint the ‘best time aggregation’—the specific combination of days that, when analyzed together, offered the strongest correlation with the final tuber yield.

When multiple measurements were combined, we employed and compared three statistical measures: sum, emphasizing the cumulative value of spectral data over time and highlighting the overall growth and development trajectory; mean, representing the average value of spectral data for a given period and demonstrating robustness to outliers; and max, denoting the highest value of spectral data within the specified period, for instance, capturing peak greenness or biomass accumulation.

We used the random forest (rf) algorithm [32] to identify the best time aggregation and statistic by fitting multiple models with all the variables (bands + VIs) against tuber yield. This involved systematically evaluating 21 time-span combinations and three summary statistics (mean, max, and sum) to determine the optimal approach for aggregating spectral data over time. The choice of algorithm was based on its capacity to overcome the problem of multicollinearity [33]. We used bootstrapping with the default characteristics of caret to train and validate the model [34] and to estimate 95% confidence intervals (2.5th and 97.5th percentiles).

In the literature, authors reduce individual spectral bands by averaging adjacent wavelengths into one spectral band (e.g., 5 nm bandwidth [15]) to diminish the multispectral dimension of the data. In this study, we carried out a data-driven approach in which we pre-processed the data by reducing its dimensionality as follows: (1) calculated Pearson correlation coefficient between each feature variable (bands and VIs) and yield (significance level = 0.05), (2) kept only variables with significant coefficients and rank them (from high to low) by their absolute correlation coefficient, and (3) performed a top-down search to keep the best features and remove redundant ones (when feature pairs had r > 0.7, we removed the least correlated with yield). This variable selection step was performed for each iteration of time aggregation and statistic to minimize redundancy and better discern the contribution of each variable to the final outcome of the model. Features were centered and scaled, and the Root Mean Squared Error (RMSE) was our objective function.

The best time aggregation, summary statistic, and filtered features were used to fit the spectral data to tuber yield on a plant basis. We used a multiple linear regression (MLR) models for two reasons: (1) linear effects are easier to quantify and describe, making it easier to separate and compare variable effects, and (2) the size of our dataset (263 samples). Features were centered and scaled to ensure they were within a similar range of values. We used the LOOCV (Leave-One-Out Cross-Validation) technique to evaluate the overall performance of the model in terms of RMSE, RMSE% (standardized by the mean yield × 100), and Root Mean Squared Error (R^2^). Linear regression assumes linearity between covariates and response variables, normality, no multicollinearity, and homoscedasticity [35]. Confidence intervals provide a measure of uncertainty around the estimated coefficients. We calculated the 95% Confidence Interval (95% CI) for the model coefficients to assess their significance, direction and strength of the association. To further explore the contribution of each variable to the model, we used the model-agnostic method of permutation feature importance implemented in the iml package [36].

## 4. Results

### 4.1. Descriptive Statistical Analysis of In Situ Data

**Figure 3:**
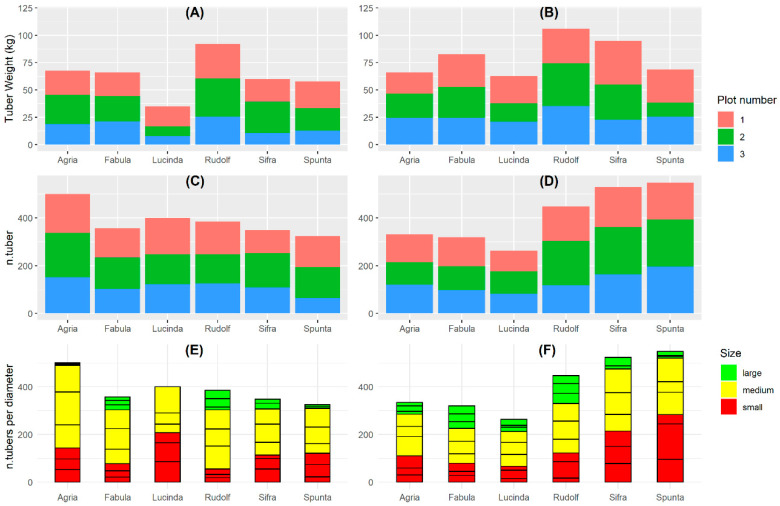
Summary statistics of tuber weight (kg), total number of tubers (n.tuber), and number of tubers according to their diameter size (>75 mm for large, 40 to 75 mm for medium, and <40 mm for small) for each experimental plot, variety, and given year. (**A**,**C**,**E**) correspond to the plots for the year 2020, and (**B**,**D**,**F**) to those for the year 2021.

Tuber yield was collected on a plant basis in six experimental plots during 2020 and 2021 (three per year) using six different potato varieties. Yields were very heterogeneous across varieties, plots and years (Figure 3). Seven plants did not have any tuber due to complete destruction from rodents. The most affected varieties (by rodents) were Sifra (24 plants), Spunta (22 plants), Agria (14 plants), Lucinda (17 plants), Rudolf (11 plants), and Fabula (8 plants). To add additional context of variability across varieties, we gathered data regarding the number and size of tubers per plant and displayed it aggregated by plots.

### 4.2. Temporal Aggregation of Spectral Data

**Figure 4:**
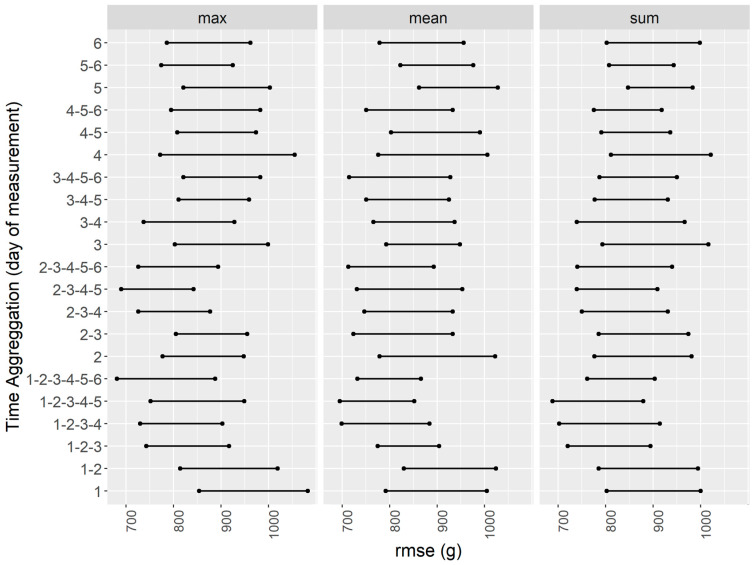
Comparison of rf results (RMSE) based on bootstrapping, expressed as 95%CI for time aggregation and statistic aggregation.

The identification of the optimal time aggregation period is crucial for predicting crop yield, as spectral data collected too early or too late in the growing season may not be informative. Figure 4 presents a comparison of model performance across different time aggregation combinations and summary statistics, highlighting the impact of timing on tuber yield prediction. Specifically, we observed that the sum statistic tended to result in higher RMSE values with less variability across time, whereas the mean and max statistics showed improved performance with the inclusion of data measured at d2. Notably, the max statistic exhibited the lowest RMSE values and narrower variability (RMSE between 690 g and 842 g), particularly when aggregating data from d2 to d5. We proceeded with the max indicator and the time-aggregation period from d2 to d5 for all subsequent analyses, as this combination demonstrated the highest predictive capability for crop yield.

### 4.3. Dimensionality Reduction and Exploratory Data Analysis

A correlation analysis was conducted to identify the most correlated and significant features influencing tuber yield while avoiding multicollinearity. Initially, the dataset comprised 1794 features (1678 individual bands + 116 vegetation indices). The first filter, based on a significance threshold (*p*-value < 0.05) for Pearson correlation coefficients (*r*), resulted in the removal of 47 features. Subsequently, the second filter, involving top-down removal of ranked features with a *r* > 0.7, led to the exclusion of 1724 additional features. This additional criterion ensured the trustworthiness of the selected variables for subsequent analyses, such as variable importance analysis. The final dataset, intended for analysis, consisted of 264 samples and 23 independent features.

Figure S1 presents a correlation matrix plot illustrating significance levels between tuber yield and the selected features. The lower triangular matrix displays bivariate scatter plots with fitted smooth lines, while the upper triangular matrix indicates Pearson correlation coefficients and their corresponding significance levels denoted by stars (* *p* < 0.05, ** *p* < 0.01, *** *p* < 0.001).

### 4.4. Model Fit and Variable Importance

**Figure 5:**
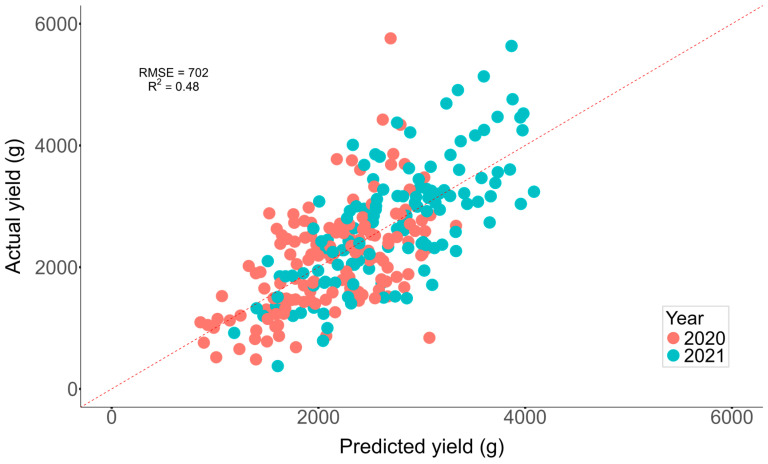
Scatter plot of measured yield and predicted yield for different potato varieties for 2020 and 2021. Points correspond to the held-out test set at each iteration of LOOCV.

**Figure 6:**
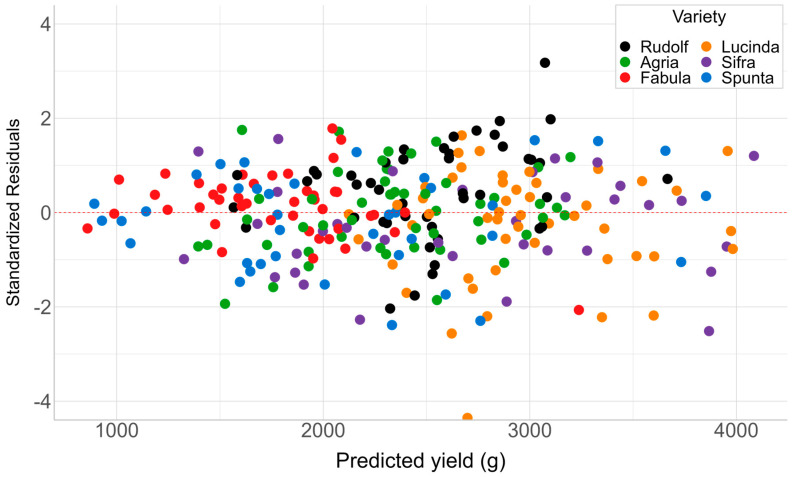
Standardized residual plot showing characteristics of varieties.

An MLR model was used to determine the relationship between a suite of independent spectral bands and VIs with respect to potato yield (on a plant basis). Table 2 shows the feature coefficients of the 23 independent variables and their corresponding 95%CI (+/−1.96 times standard error). Six coefficients showed significance (at a confidence level of 95%): CAI, CRI3, DPI, Gitelson2, PRI, and Vogelman. The model performance metrics calculated using the held-out samples of LOOCV were RMSE = 702 g and %RMSE = 29.2% (estimates of the absolute and relative error), and R^2^ = 0.48 (variance explained by the model). Figure 5 visually represents the predicted yield versus the actual yield, and Figure 6 represents the standardized residuals.

The model tends to overestimate yields for plants with actual yields below 2000 g and underestimates those surpassing 4000 g. The latter is more prominent and could be attributed to the relative rarity of such high-yielding plants, leading to limited representation during the model fitting and potentially rendering the model less robust in handling these infrequent samples. Interestingly, we observed that this trend was consistent across both years of record, with no significant differences in the model’s performance for high-yielding plants between 2020 and 2021, except for very few samples (e.g., 2020, actual weight > 5000 g/plant). This suggests that the model’s tendency to underestimate high yields is not specific to one year or a set of conditions but rather a general limitation of the model. Furthermore, an assessment of the model’s stability and potential biases was conducted through the examination of the residual plots (Figure 6). The residuals, representing the difference between model-predicted and actual yields, were standardized to express them in standard deviation units. The residuals displayed randomness and a uniform distribution across varieties. However, for plants with lower predicted yields, residuals were closer to zero.

**Figure 7:**
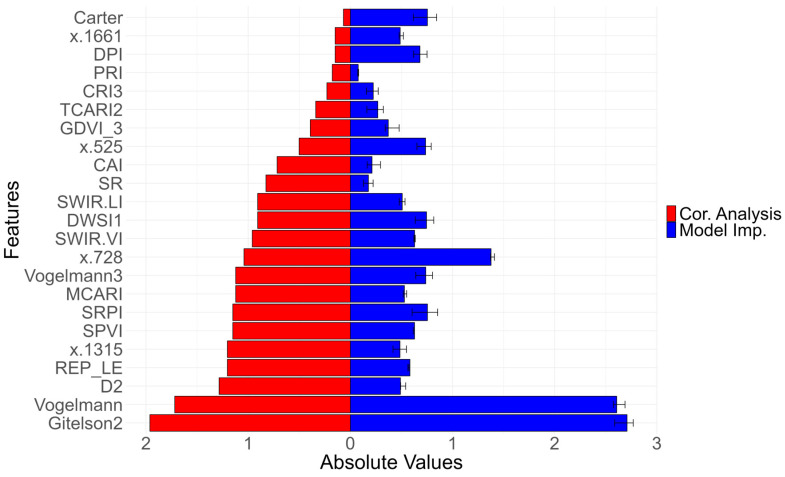
Variable importance expressed in standardized units (centered and scaled). Y-axis shows the selected variables after the dimensionality reduction process. X-axis shows, in absolute values, the standardized scores of (red) absolute Pearson correlation coefficients with yield, and (blue) importance scores from MLR model using permutation feature importance and 95% CI error bars.

Finally, we explored the insights derived from the importance of permutation features, which evaluate the significance of a feature by measuring the increase in loss when the feature is permuted. We confronted the ranked variables against the correlation analysis (Figure 7). In the latter analysis, a key emphasis was placed on identifying variables that exhibit a high correlation with the target variable while aiming to avoid multicollinearity with a setup threshold (*r* > 0.7). Notably, the two most correlated variables align closely with those identified as the most important by the MLR model, namely Gitelson2 and Vogelmann. This convergence underscores a consistent and reliable identification of influential predictors while including a rigorous consideration of multicollinearity elimination. The third most important variable in the MLR model (permutation method) was the reflectance band at 728 nm (red-edge). However, its coefficient was non-significant (Table 2).

## 5. Discussion

This study aimed to determine the optimal temporal aggregation for canopy signal measurement to predict crop yield and identify key spectral bands and VIs using hyper-spectral reflectance data.

Previous investigations into temporal aggregation in remote sensing by scholars such as [37] emphasize the usefulness of capturing the temporal evolution of crop growth and environmental conditions. In our study, we used the max statistic from 56 to 100 days after planting (d2 to d5) to temporally cover the phases of initial growth rate, leaf development, the mid-season’s maximum greenness, and photosynthetic capacity during stages like inflorescence emergence, flowering, and tuber bulking. This approach involved selecting the highest spectral data value within the specified period.

Our approach, involving correlation analysis and dimensionality reduction, identified 23 independent features significantly correlated with tuber yield. Several studies underscore the significance of feature selection and correlation analysis in agricultural remote sensing to cope with data complexity, noise, and redundancy or to improve model performance [38,39]. The most important features in the MLR model were Gitelson2 [40] and Vogelmann [41].

These VIs and bands are pivotal for estimating essential biophysical and biochemical properties of plants (e.g., chlorophyll and leaf water content), providing key insights into photosynthetic capacity, water stress, and overall health. Gitelson2 has been specifically linked to chlorophyll content, which can provide information about photosynthesis and drought resistance [42] and, consequently, crop yield. Vogelmann index focuses on the red-edge region, which serves as a valuable indicator for assessing leaf chlorophyll and water content, salinity stress, or leaf solar geometry [17,43,44]. These indices strategically leverage specific wavelengths, within the visible (red) and NIR regions, where critical information about plant health and photosynthetic activity is obtained. By exploiting the absorption of red light for photosynthesis and the reflection of NIR light from leaf cell structures, they offer valuable insights into fundamental plant biophysical properties. The physical relevance of these selected indices in gauging and forecasting crop productivity lies in their ability to capture and interpret these crucial biochemical and biophysical plant characteristics.

The permutation feature importance also showed the band’s relevance at 728 nm. Studies such as [45] those highlighting the importance of the red-edge region in estimating nitrogen concentration in biomass. Red-edge bands have been widely used to estimate canopy chlorophyll and nitrogen [46,47], and biophysical parameters related to crop yield, such as LAI [48]. However, the interpretation of the model coefficients (Table 2) shows that this band was statistically insignificant.

In this study, the impact of rodents posed a challenge. Unlike other crop pests and diseases, where alterations in crop reflectance could be detected [49], the impact of rodents caused low yields without clear manifestations in above-ground reflectance. Thus, we decided to discard plants affected by rodents from the analysis. Residual analysis revealed a homogeneous distribution across varieties, indicating no bias toward any specific one. This supports the model’s versatility, as it is not influenced by varietal specificity. Despite inherent differences in productivity, maturity time, and resilience to various stresses (e.g., water deficit), the model-related reflectance to yield with RMSE = 702 g and %RMSE = 29.2%. To the best of our knowledge, this is the first attempt to assess the relationship of ASD hyperspectral data (bands and VIs) with potato yield on a per-plant basis using different varieties. Our analysis using a linear model reveals a significant correlation between the Gitelson 2 and Vogelmann indices and potato yield. These indices, which infer the biophysical and biochemical properties of plants, can serve as reliable indicators of the plant’s health and productivity.

The main limitation of this study is the sample size, which may affect generalizability, and the exclusive focus on specific varieties limits broader applicability. Caution is needed when extending findings beyond the specific varieties studied. In addition, the reliance on spectral data may overlook environmental or soil influences, although they may be inferred from spectral data. Our results suggest the potential for even more robust outcomes with extended study periods, increased soil type diversity, and more varieties. The late sowing date in 2020 was caused by mobility restrictions due to the COVID-19 pandemic.

## 6. Conclusions

In this study, we identified the optimal time window for measuring canopy reflectance and the most sensitive spectral data related to potato yield. To gather insights into the overall health and condition of the plants, we employed a handheld ASD for hyperspectral data retrieval. The interplay of radiation with crop canopies is influenced by factors such as leaf biochemical composition, physical structure, canopy density, soil, and environmental background. Our methodology, involving meticulous filtering and testing multiple spectral bands and VIs, supports the recommendation to include the Gitelson2 index and Vogelmann index to build potato yield models based on spectral data.

This information holds great potential for designing purpose-specific sensors, with potential applications in efficient drone-based systems or broader spatial coverage and enhanced temporal resolution in satellite-based systems.

## Figures and Tables

**Table 1:** Data related to the sowing and harvest dates, planting conditions, and dates of field ASD measurements were expressed in days after planting (DAP).

Year	2020	2021
Date of sowing	25 May	9 April
Plantation depth	15–20 cm	15–20 cm
Separation between plants (same row)	60 cm	60 cm
Separation of rows	75 cm	75 cm
ASD measurements (DAP)	46, 56, 86, 93, 100, and 107	55, 62, 81, 91, 98, 109, and 118
Date of harvest	14 September	12 August

**Table 2:** Regression coefficients, 95%CI, and their significance at a confidence level of 95% (*) for the generated model. The variable coefficients are reported in their original scale.

	Coefficients	95%CI
(Intercept)	2406.5	[2317.3, 2495.8]
x.525	25.0	[−162.3, 212.4]
x.728	273.1	[−88.0, 634.1]
x.1315	−205.1	[−455.7, 45.6]
x.1661	92.2	[−150.7, 335.1]
CAI	132.8 *	[20.2, 245.5]
Carter	−5.6	[−202.4, 191.3]
CRI3	−181.2 *	[−316.0, −46.3]
D2	91.6	[−25.0, 208.2]
DPI	−221.7 *	[−434.0, −9.4]
DWSI1	−17.9	[−156.8, 121.1]
GDVI_3	−110.9	[−261.6, 39.7]
Gitelson2	−353.3 *	[−613.0, −93.6]
MCARI	83.8	[−118.8, 286.4]
PRI	158.1 *	[13.0, 303.3]
REP_LE	−75.1	[−169.9, 19.7]
SPVI	−64.7	[−417.4, 288.1]
SR	138.6	[−63.1, 340.3]
SRPI	0.9	[−163.6, 165.3]
SWIR.LI	90.2	[−152.9, 333.3]
SWIR.VI	−63.8	[−226.7, 99.2]
TCARI2	−185.9	[−406.3, 34.6]
Vogelmann	350.0 *	[81.8, 618.1]
Vogelmann3	21.9	[−156.9, 200.7]

## Data Availability

No new data were created or analyzed in this study.

## Supplementary Materials

The following supporting information can be downloaded at: https://www.mdpi.com/article/10.3390/plants13233436/s1, Figure S1: Correlation matrix of tuber yield and selected features with Pearson correlation coefficients and significance levels (* *p* < 0.05, ** *p* < 0.01, *** *p* < 0.001).
