# Supplementary figures and images for: Detecting Sensitive Spectral Bands and Vegetation Indices for Potato Yield Using Handheld Spectroradiometer Data
Source: Plants (Basel). 2024 Dec 7;13(23):3436. doi: 10.3390/plants13233436 (PMC11644505; doi:10.3390/plants13233436)

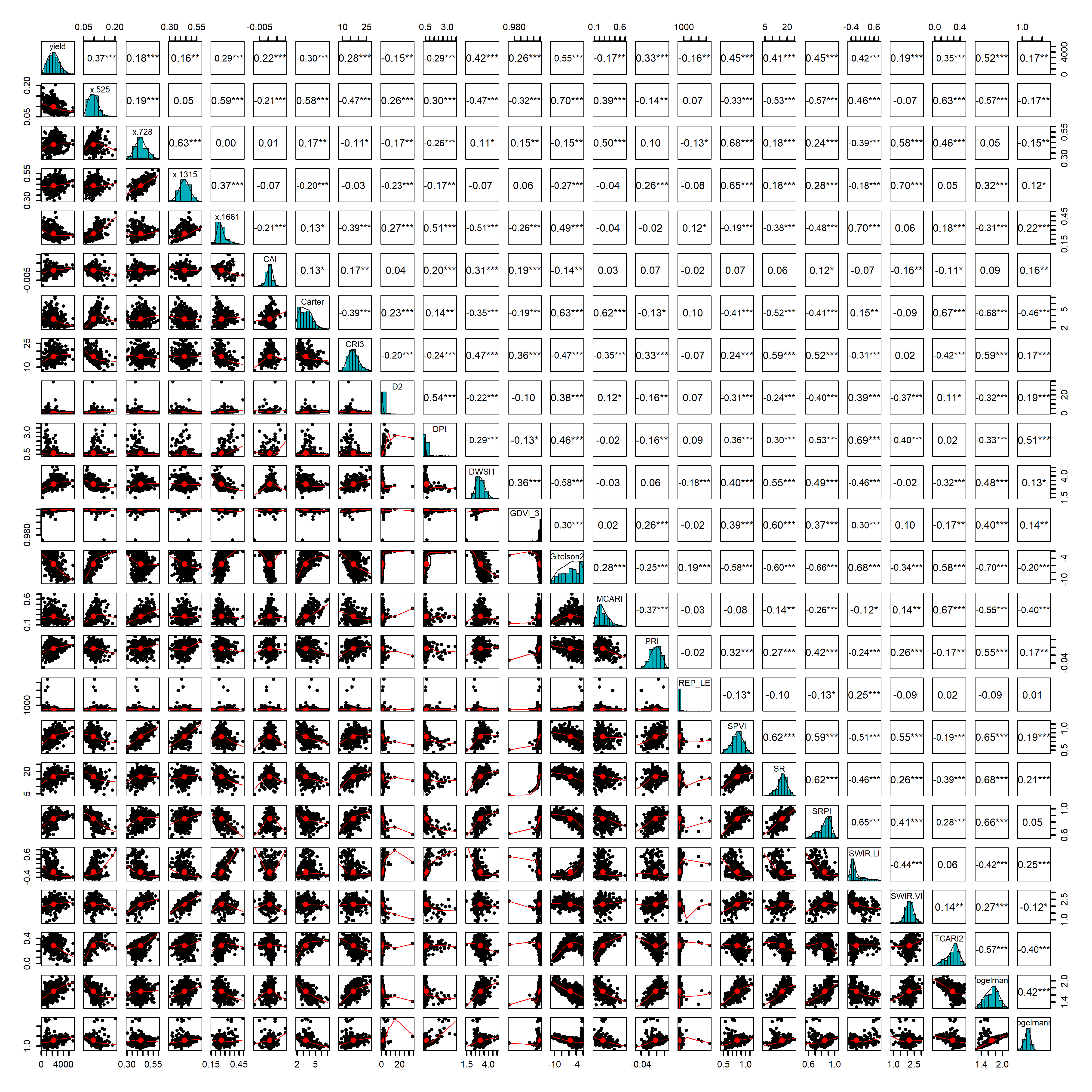

Supplement: Supplementary file 1 [file plants-13-03436-s001.zip › plants-3350367-supplementary.tiff]
